# Prognostic factors and outcomes in anti-neutrophil cytoplasmic antibody-associated glomerulonephritis: A retrospective single-center study from southern China

**DOI:** 10.12669/pjms.40.6.9683

**Published:** 2024-07

**Authors:** Lanxing Zhong, Ying Zhang, Nan Jia, Xiaotong Su, Menger Zhong, Chunmei Zhao

**Affiliations:** 1 Lanxing Zhong Dept. of Nephrology and Rheumatology, Shenzhen Hospital, Southern Medical University, Shenzhen, China; 2 Ying Zhang Department of Nephrology, Nanfang Hospital, Southern Medical University, Guangzhou, China; 3 Nan Jia Department of Nephrology, Nanfang Hospital, Southern Medical University, Guangzhou, China; 4 Xiaotong Su Dept. of Nephrology and Rheumatology, Shenzhen Hospital, Southern Medical University, Shenzhen, China; 5 Menger Zhong Dept. of Health Management, Dongguan People’s Hospital, Southern Medical University, Dongguan, China; 6 Chunmei Zhao Dept. of Nephrology and Rheumatology, Shenzhen Hospital, Southern Medical University, Shenzhen, China

**Keywords:** Antineutrophil cytoplasmic antibody-associated glomerulonephritis (ANCA-GN), renal pathology, Prognosis, End-stage renal diseases (ESRD), Overall survival

## Abstract

**Objective:**

To investigate the prognostic factors and outcomes in patients with anti-neutrophil cytoplasmic antibody-associated glomerulonephritis (ANCA-GN) in Southern China

**Methods:**

A retrospective analysis of medical records of patients with ANCA-GN admitted to Shenzhen Hospital of Southern Medical University and Nanfang Hospital of Southern Medical University between September 2011 and September 2021 was performed. The clinical presentation, biological, and renal pathology were collected. In addition, the risk factors for end-stage renal disease (ESRD) and short-term overall survival in patients with ANCA-GN were analyzed.

**Results:**

A total of 93 patients with ANCA-GN were included in the study. Of them, 91.4%, were perinuclear anti-neutrophil cytoplasmic antibodies (MPO-pANCA)-positive. Approximately one-quarter (24.7%) of patients had progressed to ESRD, and 7.5% died within six months. Most patients presented with hematuria (94.6%), proteinuria (78.5%), elevated serum creatinine (86.0%), anemia (90.3%), and increased erythrocyte sedimentation rate (ESR) (44.1%). The majority (94.6%) of patients presented with crescent formations at histopathological examination. Serum creatinine, hemoglobin, and Birmingham vasculitis activity score (BVAS) were all independent factors for ESRD (P<0.05). Moreover, while ANCA renal risk score (ARRS) has an impact on prognosis of nephropathy, it did not influence ESRD independently (P>0.05). The effect of Berden’s histopathologic classification on ESRD has not been confirmed. Age at onset, ESR and cardiovascular involvement were all independent factors affecting short-term overall survival of patients with ANCA-GN (P<0.05).

**Conclusions:**

Serum creatinine, hemoglobin, and BVAS were all independent risk factors of ESRD, while ARRS and Berden’s histopathologic classification were not. Age at onset, ESR, and cardiovascular involvement were independent risk factors for the overall six-month survival rate in patients with ANCA-GN.

## INTRODUCTION

Anti-neutrophil cytoplasmic antibody (ANCA)-associated vasculitis (AAV) is an autoimmune disorder and characterized by relapsing and remitting necrotizing pauci-immune vasculitis that mainly affects kidneys and lungs.^1^,^2^ In case of kidney involvement, ANCA-associated glomerulonephritis (ANCA-GN) refers to AAV-induced kidney injury, which can be divided into myeloperoxidase (MPO)-ANCA-GN and proteinase-3 (PR3)-ANCA-GN.^3^ The treatment of patients with AAV is improving. However, the mortality rate for patients with AAV is still 2.7 times higher than that of the general population, with most patients progressing to end-stage renal disease (ESRD).^4^

Recent studies showed that genetic polymorphism, age, renal function at the time of diagnosis, anemia, erythrocyte sedimentation rate (ESR), C-reactive protein (CRP), complement, urine protein and ANCA affect the prognosis of AAV and ANCA-GN.^5^-^7^ Birmingham vasculitis activity score (BVAS) is often employed by clinicians to assess disease activity and prognosis.^8^ Additionally, vasculitis damage index (VDI) is used to evaluate chronic lesions with organ involvement.^9^ The predictive value of renal pathology for the prognosis of patients with ANCA-GN, particularly for ESRD, has been recognized by most experts.^10^ Nevertheless, its value in assessing the overall prognosis of the disease is still controversial.

Multiple studies have demonstrated that Berden’s histopathologic classification is a reliable predictor of ESRD risk in patients with ANCA-GN.^10^,^11^ However, few data are available about how ANCA renal risk score (ARRS), proposed in 2018,^12^ correlates with outcomes of ANCA-GN. According to a recent study,^13^ ARRS has a better predictive value for ESRD than the histopathological classification, and a higher score is associated with a significantly higher risk of progression to ESRD. However, the study had no personalized treatment data to allow for a modeling of the association with renal outcomes.

This retrospective study aimed to investigate the predictive value of renal pathology and clinical presentation in evaluating nephropathy outcomes and mortality in patients with ANCA-GN in southern China who were assessed using the ARRS, BVAS and VDI scoring systems.

## METHODS

This retrospective cohort study included patients with ANCA-GN from Shenzhen Hospital of Southern Medical University or Nanfang Hospital of Southern Medical University between September 1^st^, 2011 and September 1^st^, 2021.

### Inclusion and Exclusion Criteria:

All AAV patients met the diagnostic criteria of Chapel Hill Consensus Conference 2012 (CHCC 2012) and had renal involvement, including abnormal urine test or elevated serum creatinine.^14^ And patients with AAV pauci-immune glomerulonephritis diagnosis confirmed by kidney biopsy were eligible. Patients with secondary vasculitis (such as vasculitis secondary to drugs, Henoch-Schonlein purpura, neoplasms, systemic lupus erythematosus, rheumatoid arthritis, and infection) or combined with other renal diseases (such as membranous nephropathy, diabetic nephropathy, hypertensive nephropathy) were excluded.

### Ethical Approval:

The study received ethical approval from the Joint Research Ethics Committee, Shenzhen Hospital, Southern Medical University (Approval No: NYSZYYEC20210030 October 23, 2021)

### Data collection:

Clinical, biological, and histological data were retrospectively collected from medical records. Patient characteristics such as age, sex, serotype of ANCA, serum creatinine (Scr) levels (µmol/L), height (m), weight (kg), proteinuria, hematuria, uric acid (UA) (μmol/L), hemoglobin (g/L), serum albumin (Alb) (g/L), ESR (mm/h), CRP (mg/L), complement, Berden’s histopathologic classification, BVAS, VDI, ARRS, renal pathology, combined disease and extrarenal clinical presentation. There was a six months observation period between the time of diagnosis and ESRD or death.

### Grouping and partial definitions:

The outcome of ANCA-GN were death or ESRD. Patients were grouped based on the mortality outcome (death or survival), based on the disease progression (ESRD- and a non-ESRD groups). Berden’s histopathologic classification results classified patients into focal, crescentic, mixed and sclerotic classes according to the ratio of normal glomerulus, crescentic glomerulus and spherical glomerular sclerosis.^11^ ARRS was graded based on the conditions of normal glomeruli, renal tubular atrophy and interstitial fibrosis, and glomerular filtration rate at the time of diagnosis and divided into low-, medium- and high-risk groups according to the scores.^12^ ESRD was defined as chronic dialysis or kidney transplantation (>90 days).

### Statistical analysis:

SPSS 25.0 was used for statistical analysis. The normally distributed continuous data were presented as mean ± standard deviation (SD) and compared using the Student’s t test. The non-normally distributed continuous data were presented as median [interquartile range (IQR)], and compared using the Mann-Whitney U test. Categorical variables were presented as number (percentage) and compared with the χ^2^ test. Comparisons between groups were performed using log-rank tests, and odds ratios (ORs) and 95% confidence intervals (CIs) were calculated. Cox regression analysis was used to calculate the hazard ratio (HR) of clinical presentation and histopathology in overall survival in the short term after adjustment. *P* values <0.05 were considered statistically significant.

## RESULTS

As summarized in Table-I, of 93 patients (43 males and 50 females), 23 patients (24.7%) progressed to ESRD, and 7 patients (7.5%) died. The median age of onset was 58 years. Sixty-six patients (70.1%) had involvement of the respiratory system, 17 patients (18.3%) had involvement of the cardiovascular system, 57 patients (61.3%) had combined coinfection, and 38 patients (40.9%) had combined hyperuricemia. Baseline Scr was 287.0 μmol/L (IQR 135.5-518.0). The baseline BVAS was 17 (IQR 14-19). The mean hemoglobin level was 87.0±19.6 g/L, and the mean ESR was 86.6±28.2 mm/h. Eighty-five patients (91.4%) displayed MPO-ANCA positivity (Table-I).

**Table-I:** General characteristics of patients with ANCA-GN.

Variable	Group	(Mean±SD)/ Median(IQR)/ N (%)	Variable	Group	(Mean±SD)/ Median(IQR)/ N (%)
BMI (kg/m^2^)		21.61±3.50	Scr (μmol/L)		287(135.5,518)
Onset Age (year)		58(46,64)	Alb (g/L)		31.38±6.59
Gender	Male	43(46.2)	ESR (mm/h)		86.57±28.16
	Female	50(53.8)	CRP (mg/L)		19.06(7.1,70.1)
BVAS score		17(14,19)	C3 (g/L)		1.08(0.94,1.24)
VDI score		4(2,5)	Hemoglobin (g/L)		87.02±19.63
ARRS group	Low	15(16.1)	Berden’s classification	Focal class	19(20.4)
	Medium	41(44.1)		Crescentic class	28(30.1)
	High	37(39.8)		Mixed class	29(31.2)
Nephrotic syndrome	Yes	6(6.5)		Sclerotic class	17(18.3)
	None	87(93.5)	Number of crescents		6(3,17)
Chronic nephritis syndrome	None	70(75.3)	Arteriolar necrosis	None	88(94.6)
Acute kidney injury	Yes	21(22.6)		Yes	5(5.4)
	None	72(77.4)	Renal arteriolar stenosis	None	8(8.6)
Chronic renal insufficiency	Yes	32(34.4)		Yes	85(91.4)
	None	61(65.6)	Fuchsinophilic protein deposition	None	85(91.4)
Acute exacerbation of CKD	Yes	9(9.7)		Trace	8(8.6)
	None	84(90.3)	Electron dense deposition	None	88(94.6)
Rapidly progressive nephritis	Yes	21(22.6)		Mild	4(4.3)
	None	72(77.4)		Moderate	0(0)
Coinfection	None	36(38.7)		Severe	1(1.1)
	Yes	57(61.3)	Infiltration of renal interstitial Inflammatory cells	None	3(3.2)
Hyperuricemia	None	55(59.1)		Mild	17(18.3)
	Yes	38(40.9)		Moderate	48(51.6)
Respiratory system involvement	None	27(29)		Severe	25(26.9)
	Yes	66(71)	Normal glomerular ratio		
Cardiovascular involvement	None	76(81.7)	Renal interstitial fibrosis	<25%	10(10.8)
	Yes	17(18.3)		25-50%	10(10.8)
Hematuria	+	88(94.6)		50-75%	20(21.5)
	-	5(5.4)		≥75%	53(57)
Semi-quantitative urine protein	-	20(21.5)	Renal tubular atrophy	None	11(11.8)
	±	0(0)		<25%	12(12.9)
	1+	32(34.4)		25-50%	22(23.7)
	2+	27(29)		50-75%	48(51.6)
	3+	14(15.1)		≥75%	0(0)

Values were expressed as mean ± SD for normally distributed data and median with interquartile range for non-normally distributed data, or n (%) for categorical variables. BMI, body mass index; Scr, serum creatinine; Alb, albumin; ESR, erythrocyte sedimentation rate; CRP, C-reactive protein; BVAS, Birmingham vasculitis activity score; VDI, vasculitis damage index; ARRS, ANCA renal risk score; CKD: chronic kidney disease.

### Renal survival:

Overall, renal survival was 75.3%, and ESRD was 24.7% in the first six months after the diagnosis. In terms of age, sex, body mass index (BMI), ANCA serotype, ESR, CRP, and hematuria, there was no significant difference between the ESRD and non-ESRD groups (Table-II). BVAS, VDI, and ARRS scores were higher in ESRD patients (p<0.01). ESRD patients had worse renal function at baseline and more frequently experienced hyperuricemia (p=0.024). ESRD was associated with less frequent chronic nephritis syndrome (p=0.009). Moreover, the incidence of rapidly progressive nephritis was lower in the ESRD patients. ESRD patients had more serious anemia and lower C3 (p<0.05).

**Table-II:** Clinical presentation of patients with ANCA-GN between ESRD and non-ESRD groups [n(%)]

Variable	Non-ESRD	ESRD	χ^2^/t/z	P
BMI (kg/m^2^)	21.28±3.49	22.6±3.42	-1.57	0.12
Onset Age (year)	57(46,63)	59(45,73)	-1.198	0.231
Gender			0.433	0.510
Male	31(44.3)	12(52.2)		
Female	39(55.7)	11(47.8)		
BVAS score	16.5(13,19)	18(17,21)	-2.664	0.008
VDI score	3(2,5)	5(4,6)	-2.791	0.005
ARRS group			9.255	0.010
Low	14(20)	1(4.3)		
Medium	34(48.6)	7(30.4)		
High	22(31.4)	15(65.2)		
Nephrotic syndrome	5(7.1)	1(4.3)	0	1
Chronic nephritis syndrome	22(31.4)	1(4.3)	6.820	0.009
Acute kidney injury	16(22.9)	5(21.7)	0.012	0.911
Chronic renal insufficiency	23(32.9)	9(39.1)	0.302	0.583
Acute exacerbation of CKD	8(11.4)	0(0)	0.348	0.555
Rapidly progressive nephritis	12(17.1)	9(39.1)	4.788	0.029
Coinfection	40(57.1)	17(73.9)	2.052	0.152
Hyperuricemia	24(34.3)	14(60.9)	5.063	0.024
Respiratory system involvement	50(71.4)	16(69.6)	0.029	0.864
Cardiovascular involvement	13(18.6)	4(17.4)	0	1
Alb (g/L)	31.84±6.62	29.99±6.44	1.17	0.245
Semi-quantitative urine protein			3.92	0.268
-	16(22.9)	4(17.4)		
±	0(0)	0(0)		
+	27(38.6)	5(21.7)		
++	17(24.3)	10(43.5)		
+++	10(14.3)	4(17.4)		
Scr (μmol/L)	206(114,360)	618(366,807)	-4.421	0
UA (μmol/L)	416.82±147.78	450.12±162.69	-0.914	0.363
ESR (mm/h)	86.21±27.22	87.65±31.48	-0.211	0.833
CRP (mg/L)	21.35(7.3,70.91)	17.1(5.28,56)	-0.704	0.482
C3 (g/L)	1.08(0.96,1.26)	0.99(0.88,1.1)	-2.004	0.045
Hemoglobin (g/L)	90.51±20.1	76.39±13.68	3.135	0.002

Values were expressed as mean ± SD for normally distributed data and median with interquartile range for non-normally distributed data, or n (%). Differences among the groups were analyzed by Student’s t test for normally distributed values and by the Mann-Whitney U test for nonparametric values. Pearson’s χ^2^ test was employed to analyze categorical data. AAGN, anti-neutrophil cytoplasmic antibody-associated glomerulonephritis; BMI, Body mass index; BVAS: Birmingham Vasculitis Activity Score; VDI, Vasculitis damage index; ARRS, ANCA renal risk score; CKD, chronic kidney disease; ESR, erythrocyte sedimentation rate; CRP, C-reactive protein; Scr, serum creatinine; UA, uric acid.

We detected a trend for a higher ARRS in patients with ESRD, as shown in Table-III (*P*<0.05). Furthermore, Berden’s histopathologic classification was comparable in non-ESRD and ESRD patients (*P*>0.05). Histopathological analysis showed significantly more *fuchsinophilic* protein depositions in the ESRD group of patients compared to the non-ESRD group (*P*=0.01).

**Table-III:** Renal Pathology in ESRD and non-ESRD groups [n(%)].

Variable	Non-ESRD	ESRD	χ^2^/t/z	P
Berden’s classification			3.710	0.295
Focal class	16(22.9)	3(13)		
Crescentic class	18(25.7)	10(43.5)		
Mixed class	24(34.3)	5(21.7)		
Sclerotic class	12(17.1)	5(21.7)		
Number of crescents	6(3,15)	7(3,25)	-1.218	0.223
Arteriolar necrosis	4(5.7)	1(4.3)	0	1
Renal arteriolar stenosis	63(90)	22(95.7)	0.168	0.682
Fuchsinophilic protein deposition			6.708	0.010
No	67(95.7)	18(78.3)		
Small amount	3(4.3)	5(21.7)		
Electron-dense deposition			2.830	0.243
None	67(95.7)	21(91.3)		
Mild	3(4.3)	1(4.3)		
Moderate	0(0)	0(0)		
Severe	0(0)	1(4.3)		
Infiltration of renal interstitial Inflammatory cells			1.859	0.602
None	3(4.3)	0(0)		
Mild	13(18.6)	4(17.4)		
Moderate	36(51.4)	12(52.2)		
Severe	18(25.7)	7(30.4)		
Normal glomerular ratio	17.35(6.25,43.48)	10(2.78,33.33)	-1.797	0.072
Renal interstitial fibrosis			1.885	0.597
<25%	8(11.4)	2(8.7)		
25-50%	9(12.9)	1(4.3)		
50-75%	15(21.4)	5(21.7)		
≥75%	38(54.3)	15(65.2)		
Renal tubular atrophy			2.310	0.511
0	9(12.9)	2(8.7)		
<25%	10(14.3)	2(8.7)		
25-50%	18(25.7)	4(17.4)		
50-75%	33(47.1)	15(65.2)		

Values were expressed as mean ± SD for normally distributed data and median with interquartile range for non-normally distributed data, or n (%). Differences among the groups were analyzed by Student’s t test for normally distributed values and by the Mann-Whitney U test for nonparametric values. Pearson’s χ^2^ test was employed to analyze categorical data.

Multivariate logistic regression analysis results are shown in Table-IV and Table-V. Both blood creatinine and hemoglobin remained significant risk factors for ESRD occurrence. Higher serum creatinine levels or lower hemoglobin levels were associated with higher likelihood of disease progression to ESRD. In addition, only multivariate logistic regression analysis with a full entry model showed that BVAS score (OR=1.244, 95% *CI*: 1.005-1.540) was an independent risk factor for ESRD (Table-IV). Likewise, only the multivariate logistic regression model using a stepwise regression method showed that fuchsinophilic protein deposition [HR 26.313 (95% CI 1.695-408.49)] was an independent risk factor for ESRD: higher rate of fuchsinophilic protein depositions correlated with more likely progression to ESRD (Table-V).

**Table-IV:** Multivariate logistic regression analysis with full entry model in patients with ANCA-GN between ESRD and non-ESRD groups.

	B	S.E.	Wald	p	OR	95% CI	

						Lower limit	Upper limit
BVAS score	0.218	0.109	4.009	0.045	1.244	1.005	1.540
VDI score	-0.461	0.295	2.441	0.118	0.630	0.353	1.125
ARRS group	0.278	0.672	0.171	0.679	1.321	0.354	4.932
Chronic nephritis syndrome	-0.045	1.272	0.001	0.972	0.956	0.079	11.556
Rapidly progressive nephritis	-1.280	0.834	2.359	0.125	0.278	0.054	1.424
Hyperuricemia	1.454	0.791	3.383	0.066	4.281	0.909	20.162
Scr	0.004	0.002	5.302	0.021	1.004	1.001	1.007
C3	-0.934	1.362	0.47	0.493	0.393	0.027	5.670
Hemoglobin	-0.060	0.024	6.171	0.013	0.942	0.899	0.987
FP deposition	3.457	1.963	3.102	0.078	31.726	0.677	1486.319

BVAS, Birmingham Vasculitis Activity Score; VDI, vasculitis damage index; ARRS, ANCA renal risk score; Scr, serum creatinine; FP deposition: fuchsinophilic protein deposition.

**Table-V:** Multivariate logistic regression analysis with stepwise regression model in patients with ANCA-GN between ESRD and non-ESRD groups.

	B	S.E.	Wald	p	OR	95% CI	

						Lower limit	Upper limit
Scr	0.004	0.001	11.682	0.001	1.004	1.002	1.006
Hemoglobin	-0.048	0.020	5.890	0.015	0.953	0.916	0.991
FP deposition	3.270	1.399	5.462	0.019	26.313	1.695	408.490

Scr, Serum Creatinine; FP deposition, fuchsinophilic protein deposition.

### Predictors of overall outcome:

The outcome of renal survival in patients was not affected by ESR and age. However, Cox regression analysis (Table-VI) showed that age at onset, ESR and cardiovascular involvement were the risk factors for overall survival of patients with ANCA-GN within six months (*P*<0.05), and were all independent risk factors. Higher patient age, faster ESR, or the presence of cardiovascular involvement correlated with higher the risk of death. Surprisingly, ARRS and Berden’s histopathologic classification were not independent factors for overall six months survival (*P*>0.05).

**Table-VI:** Cox regression analysis of risk factors for overall survival in patients with ANCA-GN within six months.

Variable	Univariate analysis Multivariate analysis			

	HR(95%CI)	P	HR(95%CI)	P
Gender	0.808 (0.243-2.684)	0.728		
BMI	0.988 (0.821-1.187)	0.894		
Onset Age (year)	1.078 (1.012-1.149)	0.020	1.093(1.008-1.186)	0.032
BVAS score	1.087 (0.962-1.229)	0.18		
VDI score	0.920 (0.635-1.334)	0.66		
ARRS group	1.568(0.640-3.841)	0.325		
Nephrotic syndrome		0.964		
Chronic nephritis syndrome		0.225		
Acute kidney injury		0.769		
Chronic renal insufficiency		0.841		
Acute exacerbation of CKD		0.459		
Rapidly progressive nephritis		0.183		
coinfection	2.567(0.553-11.909)	0.116		
hyperuricemia	0.801(0.233-2.748)	0.724		
Respiratory system involvement	3.55(0.454-27.758)	0.227		
cardiovascular involvement	3.698(1.045-13.09)	0.043	4.679(1.034-21.162)	0.045
hematuria	2.605(0.561-12.101)	0.222		
semi-quantitative urine protein	0.698(0.374-1.304)	0.260		
Scr	1.001(0.998-1.003)	0.703		
UA	1.000(0.996-1.004)	0.903		
ESR	1.032(1.006-1.058)	0.014	1.045(1.012-1.078)	0.007
CRP	0.995(0.982-1.009)	0.479		
C3	0.762(0.087-6.64)	0.806		
Hemoglobin	0.99(0.960-1.020)	0.492		
Berden’s classification				
Focal class	1			
Crescentic class	0.985(0.138-7.035)	0.988		
Mixed class	1.89(0.379-9.413)	0.437		
Sclerotic class	0.442(0.04-4.951)	0.508		
Number of crescents	1.019(0.961-1.08)	0.529		
Arteriolar necrosis	2.128(0.262-17.312)	0.48		
Renal arteriolar stenosis	23.141(0.002-2665.41)	0.509		
Fuchsinophilic protein deposition	1.502(0.19-11.898)	0.700		
Electron dense deposition	1.249(0.319-4.891)	0.749		
Infiltration of renal interstitial Inflammatory cells	0.792(0.395-1.588)	0.512		
Normal glomerular ratio	0.987(0.960-1.015)	0.365		
Renal interstitial fibrosis	1.035(0.573-1.867)	0.910		
Renal tubular atrophy	1.032(0.577-1.846)	0.916		

BMI: Body mass index; BVAS: Birmingham Vasculitis Activity Score; VDI, Vasculitis damage index; ARRS, ANCA renal risk score; CKD, chronic kidney disease; Scr, serum creatinine; UA, uric acid; ESR, Erythrocyte sedimentation rate; CRP, C-reactive protein.

## DISCUSSION

The results of our study showed that serum creatinine, hemoglobin, and Birmingham vasculitis activity score (BVAS) were all independent factors for ESRD (P<0.05) in patients with ANCA-GN. ARRS was not independently associated with ESRD. Age at onset, ESR and cardiovascular involvement were all independent factors affecting short-term (six months) overall survival of patients with ANCA-GN. Moreover, we found that the majority of patients with ANCA-GN were MPO-pANCA positive, and 70.1% of patients had respiratory system injury, which was similar to other studies.^2^,^3^ Furthermore, patients with ANCA-GN predominantly exhibited hematuria with impaired renal function, which could be accompanied by proteinuria and increased blood pressure. Renal pathology revealed that 94.6% of patients had crescent formation, along with varying degrees of renal interstitial inflammatory cell infiltration, fibrosis, and tubular atrophy. Furthermore, 91.45% of patients had renal arteriole stenosis, while 5% had arteriolar necrosis. These renal manifestations and renal pathology are basically consistent with previous reports^1^,^11^ that show that patients with ANCA-GN typically exhibit pauci-immune glomerulonephritis with crescent formation, which may be accompanied by inflammation and cellulose necrosis of the small vascular wall. Immunofluorescence staining typically reveals no or only minor immune complex deposition.

Previous studies have showed that infection is the main cause of mortality in AAV patients within a year, followed by vasculitis activity.^1^,^2^ Our study revealed that most patients with ANCA-GN had anemia, increased ESR and increased CRP during the initial diagnosis, indicating that the majority of patients had disease activity at diagnosis. As 61.3% of patients experience infection within six months after the diagnosis, it is crucial to ensure improved assessment procedures, infection prevention methods and individualized treatment during the course of the disease, especially during the epidemic of influenza and SARS-CoV-2.^15^

Several studies have indicated that Berden’s histopathologic classification for predicting renal outcome of patients with ANCA-GN, and ARRS have advantages and disadvantages for the prognosis assessment of ANCA-GN.^11^-^13^ While some studies showed the association of Berden’s histopathologic classification with the overall survival of patients,^16^-^18^ our logistic regression analysis found that Berden’s histopathologic classification was not an independent factor for ESRD (*P>*0.5). The evaluation value of renal interstitial fibrosis (IF) or tubular atrophy (TA) can be used to forecast the prognosis of nephropathy.^19^ Brix et al.^12^ proposed that the ARRS might be employed to predict the risk of ESRD among patients with ANCA-GN. Interestingly, our results indicated that ARRS may have an impact on the prognosis of ESRD (*P*<0.05).

Furthermore, logistic regression analysis of the ESRD and non-ESRD groups showed that serum creatinine level was an independent risk factor for ESRD in patients with ANCA-GN (OR=1.004, 95% *CI*: 1.001-1.007, *P*<0.05). The GFR related to serum creatinine is one of the ARRS score components. Therefore, our results further demonstrated the effect of AARS on the prognosis of nephropathy. Compared with Berden’s histopathologic classification, ARRS may have a higher predictive value for a poor prognosis in nephropathy. However, the multivariate logistic regression analysis indicated that there was no independent relationship between ARRS and ESRD (*P>*0.05). Interestingly, our results indicated that the abundance of fuchsinophilic protein depositions in renal pathology is associated with higher probability of progression to ESRD. However, there are currently no similar reports available, and more studies are needed to validate our results before possible clinical application.

Recent studies have also revealed that the prognosis of ANCA-GN is partially influenced by anemia, ESR, CRP, age, BVAS, and VDI.^20^ In our study, hemoglobin levels and BVAS scores were both independent risk factors leading to ESRD. Patients with more severe anemia and higher BVAS scores were more likely to progress to the ESRD stage. This suggests the predictive value of these indexes for ESRD progression.

Our results revealed that age at onset, ESR and cardiovascular involvement were all independent risk factors of overall survival of patients with ANCA-GN at six months after the diagnosis. In our study, the average onset age of patients with ANCA-GN was 58 years, which was similar to other studies^3^ and higher the age of onset correlated with shorter survival time. We may speculate that this correlation is probably related to the age-related factors, such as underlying diseases, declining body condition, poor response to treatment, worse tolerance, etc. There was a correlation between the ESR and the risk of death, which might be related to the level of disease activity. Patients with cardiovascular involvement at diagnosis had a higher risk of death, which also indicates the impact of vital organ injuries on the disease prognosis.

According to this study, factors affecting ESRD prognosis and overall survival in patients with ANCA-GN were different. Berden’s classification, ARRS, BVAS, and VDI score were not independent risk factors for overall survival at six months after diagnosis. In addition, sex, urine red blood cells, and CRP had no predictive value for survival and nephropathy outcomes in patients with ANCA-GN.

### Limitations:

It includes the short follow-up time and insufficient number of enrolled patients. This may potentially affect our results and could partially explain the observed lack of predictive value of sex, urine red blood cells, and CRP. However, AAV is a multisystemic disease, and ANCA-GN is only one of the involved systems. Therefore, the extrarenal involvement system may partially affect the prognosis of patients with ANCA-GN. In addition, the disease itself, comorbidities, and side effects of treatment may also affect the overall prognosis of patients. Further studies that account for these potentially risk factors are needed.

## CONCLUSION

We identified serum creatinine, hemoglobin, and BVAS as independent risk factors of ESRD. There was no correlation between Berden’s histopathologic classification and ARRS, and ESRD progression. Age at onset, ESR, and cardiovascular involvement were independent risk factors for the overall six-month survival rate in patients with ANCA-GN. Further multicenter, large-sample studies with long follow-up should be conducted to explore additional risk factors for the prognosis of overall survival and ESRD in patients with ANCA-GN.

### Authors’ contributions:

**LZ:** Conceived and designed the study. **LZ**, **YZ**, **NJ**, **XS**, **MZ** and **CZ:** Collected the data and performed the analysis. **LZ:** Was involved in the writing of the manuscript and is responsible for the integrity of the study. All authors have read and approved the final manuscript.
